# ﻿A new species of *Arrhopalites* Börner (Collembola, Symphypleona, Arrhopalitidae) from China, with a key to the Asian species of the *caecus* group

**DOI:** 10.3897/zookeys.1102.81247

**Published:** 2022-05-20

**Authors:** Nerivania Nunes Godeiro, Feng Zhang, Bruno Cavalcante Bellini

**Affiliations:** 1 Shanghai Natural History Museum, Shanghai Science & Technology Museum, Shanghai 200041, China Nanjing Agricultural University Nanjing China; 2 Department of Entomology, College of Plant Protection, Nanjing Agricultural University, 210095, Nanjing, China Shanghai Natural History Museum Shanghai China; 3 Department of Botany and Zoology, Biosciences Center, Federal University of Rio Grande do Norte, Natal, Rio Grande do Norte, Brazil Federal University of Rio Grande do Norte Natal Brazil

**Keywords:** Appendiciphora, grassland, Katiannoidea, springtails, taxonomy

## Abstract

The second species of *Arrhopalites* from China is described and illustrated and an identification key to the Asian species of the *caecus* group is presented. *Arrhopalitesbrevicornis***sp. nov.** is the eleventh species of the *caecus* group recorded in Asia and it can be clearly differentiated by the unguiculus III with 2 internal teeth (0–1 in all other species). Also, the combination of antennae less than 2 times the size of the head, antennal segment IV without annulations, 1+1 eyes, dorsal head with 9 spines, 2+2 regular spines per side on the anal valves, circumanal chaetae without basal serrations, subanal appendage long and apically serrated, manubrium with 5+5, and dorsal dens with 16 chaetae is unique among the Asian species of the *caecus* group.

## ﻿Introduction

Arrhopalitidae Stach, 1956 comprises species of *Arrhopalites* Börner, 1906, *Pygmarrhopalites* Vargovitsh, 2009 and *Troglopalites* Vargovitsh, 2012. Currently this family gathers 141 species described worldwide, with 41 of them belonging to *Arrhopalites* (Bellinger et al. 1996–2022). Vargovitsh (2013) divided the genus into three species groups based on the ventral (anterior) dental chaetotaxy: *diversus* group, with ﻿the chaetal formula of 3, 2, 1, 1 from the apex to the basis of the structure; *caecus* group, with 3, 2, 1, 1, 1 chaetae; and *harveyi* group with 3, 2, 2, 1, 1 chaetae. This division, as well as the support for the family and genera, have not been tested yet with the use of molecular phylogenetics, which could clarify different points of view about the systematics of Arrhopalitidae internal systematics (Zeppelini 2011; Vargovitsh 2013). Also, such kind of study could verify the phylogenetic signal of the dental chaetotaxy within *Arrhopalites*, which is widely used among the Symphypleona to separate species groups, but may be, at least in few genera, an arbitrary feature to gather unrelated taxa (see Cipola et al. 2021: 37–38). Nevertheless, Vargovitsh’s groups of *Arrhopalites* currently provide clear data to quickly compare species within the genus (Vargovitsh 2013).

Despite its extensive territory, only one species of *Arrhopalites* was recorded from China so far, *A.pukouensis* Wu & Christiansen, 1997, described from Jiangsu Province, in the eastern region of the country. Another species (*A.nanjingensis* Lin & Chen, 1997) was originally described as *Arrhopalites*, but it was transferred to *Pygmarrhopalites* by Vargovitsh (2009). So, herein we describe in detail a second species of *Arrhopalites* from China and provide an identification key to the Asian species of the *caecus* group.

## ﻿Materials and methods

Specimens were collected in the field with entomological aspirators and transferred to plastic containers in the laboratory of Entomology, Nanjing Agricultural University (NJAU), China, where they are being cultured. Specimens used for description were sorted in September 2021 and transported to Shanghai Natural History Museum, where the following steps were developed. Under a stereomicroscopy Teelen XTL-207, specimens were bleached and diaphanized, first in 5% KOH and after in 10% lactophenol for three minutes/each. Hoyer’s liquid was used to mount the specimens between a slide and a glass coverslip. Slides were dried in an oven at 50 °C for 10 days (Christiansen and Bellinger 1980, 1998). A Leica DM2500 microscope with a drawing tube was used to draw the illustrations, which were posteriorly vectorized with Corel Draw 2018 v20. Habitus of the species was photographed in 70% ethanol under a Leica S8AP0 stereomicroscope attached to a Leica DMC4500 camera, using Leica Application Suite software. Slides with type specimens mounted in Hoyer’s liquid along with 78 specimens preserved in 98% ethanol are deposited at the collection of Shanghai Natural History Museum (SNHM).

The terminology used in descriptions follows Fjellberg (1999) for the labial palp papillae, Cipola et al. (2014) for the labral chaetotaxy, Nayrolles (1988) for the proximal tibiotarsi chaetotaxy, Betsch and Waller (1994) for head and anterior large abdomen chaetotaxy, Vargovitsh (2009, 2012, 2013) for the posterior large abdomen chaetotaxy and Betsch (1997) for the small abdomen chaetotaxy. On the dens we considered as the dorsal chaetae the sum of the dorsal, dorso-internal and dorso-lateral rows. Drawings and observations were made based in the entire type series.

The abbreviations used in the text and drawings are: Abd = abdominal segment(s); Ant antennal segment(s); and Th = thoracic segment(s).

## ﻿Taxonomy

**Order Symphypleona Börner, 1901 sensu Bretfeld, 1986**

**Suborder Appendiciphora Bretfeld, 1986**

**Superfamily Katiannoidea Bretfeld, 1994**

### ﻿Family Arrhopalitidae Stach, 1956 *sensu* Bretfeld, 1999

#### Genus *Arrhopalites* Börner, 1906

##### ﻿ Arrhopalites brevicornis sp. nov.

Taxon classificationAnimaliaSymphypleonaArrhopalitidae

418B7106-1BFB-5A47-91C9-1053FD32A87F

http://zoobank.org/FED6DB53-B746-424D-93B0-105DA1AFA930

**Figure 1.**
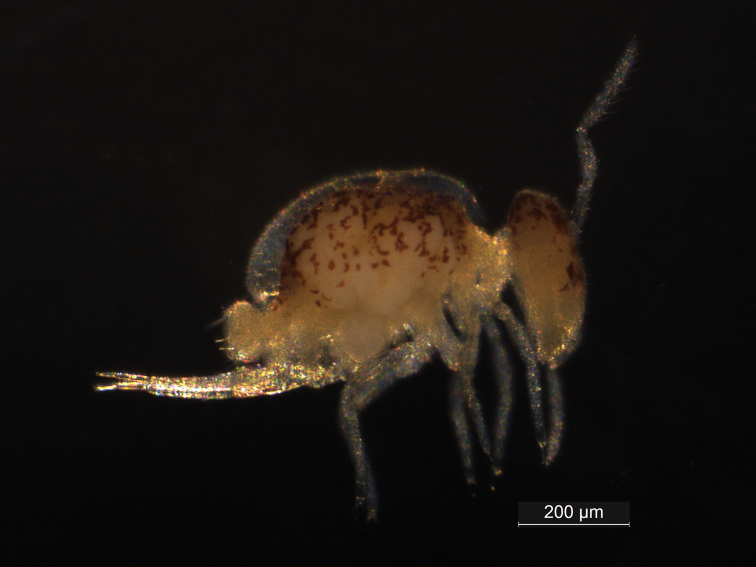
*Arrhopalitesbrevicornis* sp. nov.: habitus of specimen fixed in ethanol.

**Figure 2.**
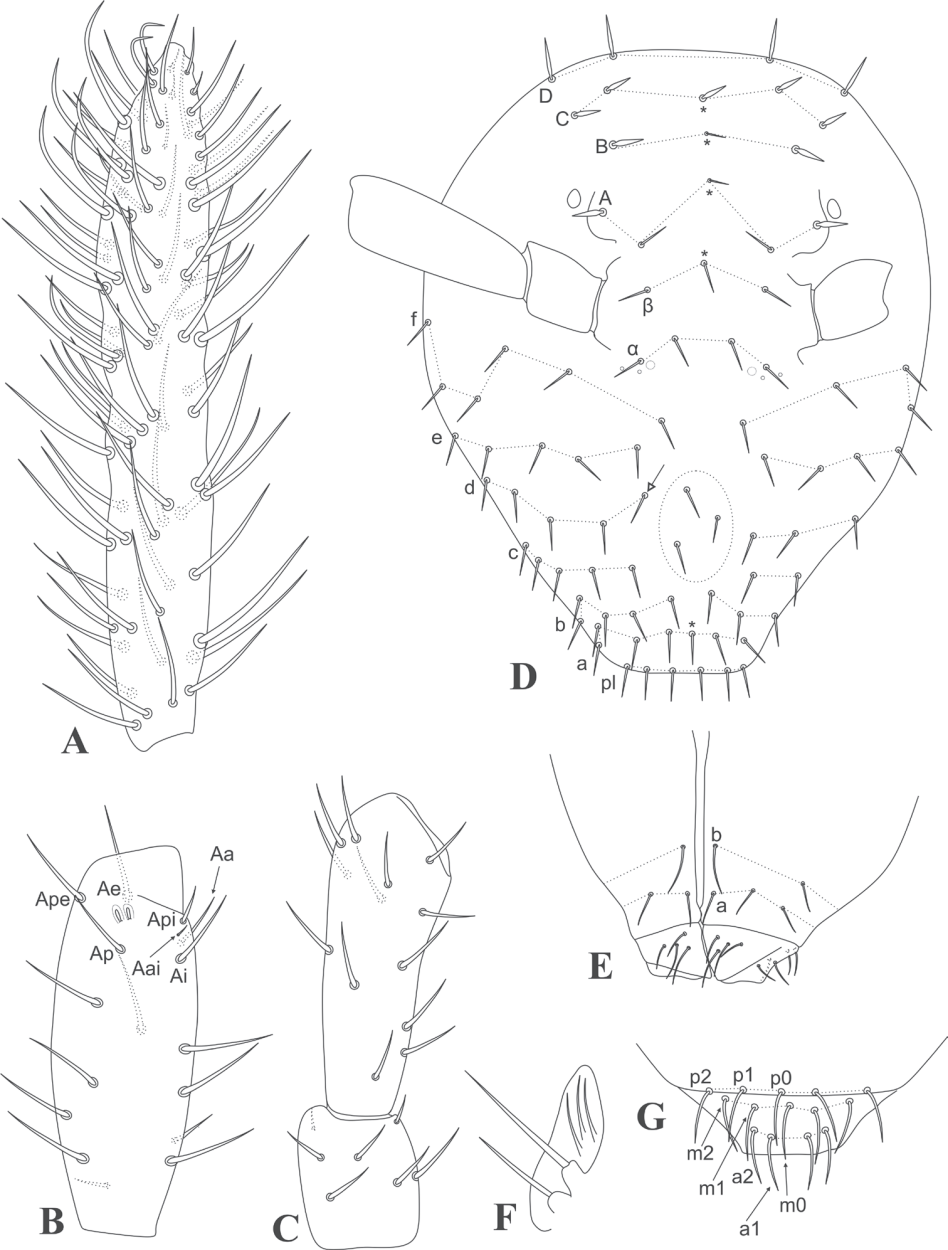
*Arrhopalitesbrevicornis* sp. nov. head **A** left Ant IV (dorsal side) **B** left Ant III (ventral side) **C** left Ant I–II (dorsal side) **D** anterior head – left side shows the complete chaetotaxy, * marks unpaired chaetae, white arrow points to chaeta present or absent, large dashed circle on central clypeal region marks asymmetrical chaetae **E** ventral head chaetotaxy – right side shows the complete chaetotaxy, including labial basomedian and basolateral fields **F** left maxillary outer lobe and sublobal plate **G** prelabral chaetae and labrum.

**Figure 3.**
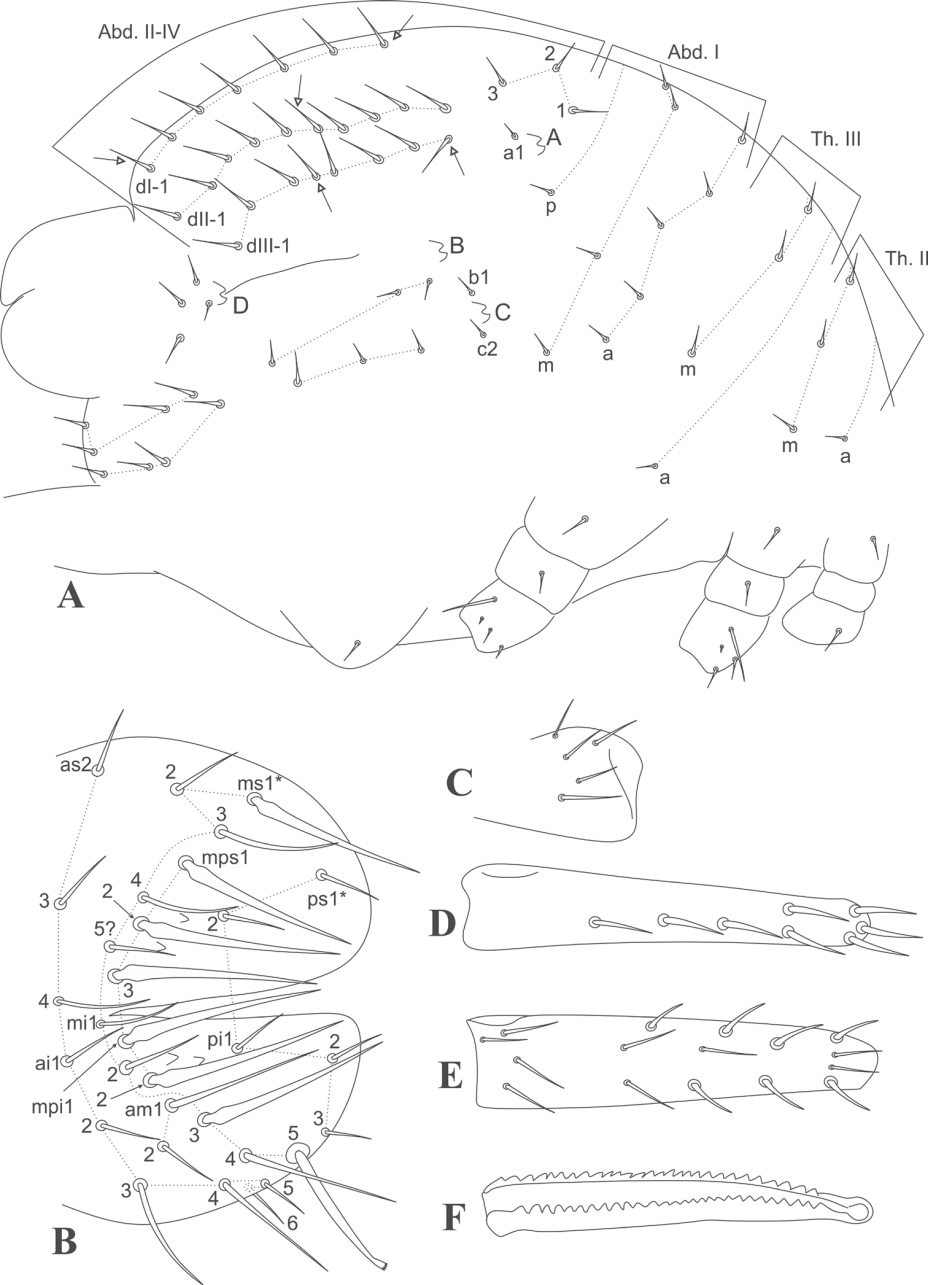
*Arrhopalitesbrevicornis* sp. nov. trunk, proximal legs and furca **A** large abdomen and coxae chaetotaxy (right side) **B** female’s small abdomen (left side) **C** right manubrial chaetotaxy (dorsal side) **D** right dens ventral chaetotaxy **E** right dens dorsal chaetotaxy **F** mucro.

**Figure 4.**
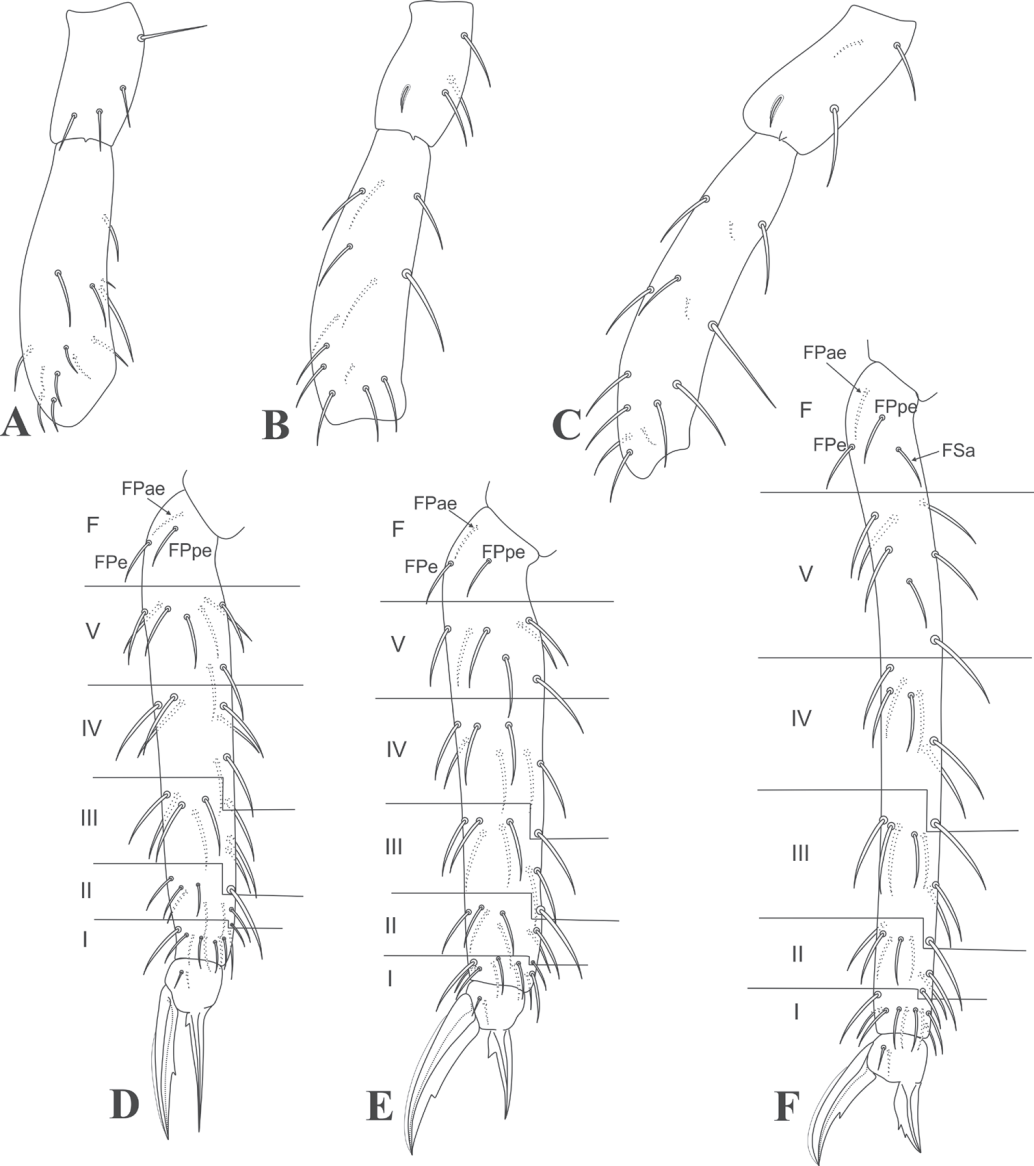
*Arrhopalitesbrevicornis* sp. nov. legs **A–C** trochanters and femurs I–III (anterior side), respectively **D–F** tibiotarsi and empodial complexes I–III (anterior side), respectively.

Figs 1 , 2 , 3 , 4 , Table 1

###### Type material.

***Holotype*** on slide “SNHM00001”: female, Jilin Province, China, 44°33'N, 123°31'E, 2013, in soil samples from the Ecological Research Station for Grassland Farm, July 2013, Bing Zhang leg. ***Paratypes*** on slides: 9 females on slides, same data as holotype. Besides the type material, 78 specimens are kept in 98% ethanol at the SNHM, plus several paratype slides are kept at the laboratory of Entomology, NJAU, China.

###### Diagnosis.

**Female.** Antennae short, about 1.4 times the head length. Ant IV not subdivided and short, about twice or less the length of Ant III. Eyes 1+1. Clypeal area a–f lines with 7(+1)/7/5/4–5/5/6 chaetae respectively, plus 3 central chaetae with unclear homologies, frontal area A–C lines with 1/1/2(+1) short stout spines. Small abdomen, dorsal anal valve with 2 cuticular spines per side and 4 sword-shaped smooth chaetae (ms1, mps1–3), ventral anal valves with 2 cuticular spines each and 3 sword-shaped smooth chaetae (mi3, mpi1–2), subanal appendage long, similar in length to mi3, mpi1–2, with a spatulated and apically serrated apex. Manubrium with 5 chaetae on each side, dens ventral formula from the apex to the basis as 3,2,1,1,1, dorsally with 16 chaetae. Mucro with both edges serrated, apically swollen. Ungues I slender, III broad, all with an underdeveloped tunica, unguiculus III with 2 inner teeth.

###### Description.

**Female**. Body (head + trunk) length of type series (females, *N* = 4) ranging between 0.71 and 0.81 mm, average 0.74 mm, holotype with 0.75 mm. Habitus as in Fig. 1. Specimens pale yellowish with brownish spots of pigment on frontal and dorsal head and dorso-lateral large abdomen. Body chaetae smooth and acuminate, with the exception of the subanal appendage.

Head (Figs 1, 2). Antennae shorter than the body, with 0.32 mm in the holotype (Fig. 1), ratio antennae: head length of the holotype 1.3:1, type series average 1.4:1. Holotype antennal segments ratio of Ant I:II:III:IV as 1:1.6:2.3:4.3, and of type series (*N* = 4) as 1:1.3–2.7:2.1–3.3:3.8–6.7. Ant IV short and stout, about twice or less the size of Ant III (in holotype, ratio Ant III:IV = 1:1.87), without subsegments, with about 87 regular chaetae of different sizes distributed in apparently 13 whorls (Fig. 2A). Ant III slightly swollen with 17 chaetae, Ape, Ae, Ap, Ai, Aa, Api, and Aai present, Api slightly reduced, Aai as the accessory microsensillum, sense rods not swollen inside separate invaginations (Fig. 2B). Ant II with 13 regular chaetae, Ant I with 7, the two more apical reduced (Fig. 2C). Eyes 1+1, head length (eyes to mouth) of holotype 0.25 mm. Clypeal area a–f lines with 7(+1)/7/5/4–5/5/6 chaetae respectively, plus 3 central chaetae of unclear homologies; interantennal area **α** and **β** lines with 2/1(+1) short chaetae respectively, plus 2+2 small oval organs (pseudopores) and 1+1 large circles lacking tegument granules near the lateral chaetae on **α** line; frontal area A–C lines with 1/1/2(+1) short stout spines, D line with 2 elongate thinner erect chaetae (Fig. 2D). Ventral groove with 2 surrounding chaetae from lines a and b, labial basomedian field with 4, basolateral field with 5 chaetae (Fig. 2E). Labial papilla E lateral finger-shaped, not reaching the papilla apex, other labial structures unclear. Maxillary outer lobe apical chaeta longer than the basal one, sublobal plate with three sublobal hairs (Fig. 2F). Distal margin of the clypeus with 3 prelabral chaetae, labral chaetotaxy with 2(+1) p, 2(+1) m and 2 a chaetae, all subequal (Fig. 2G).

Trunk (Fig. 3A, B). Trunk length of holotype 0.5 mm. Large abdomen: thorax continuous with abdomen, without any constrictions. Th II with 1 a and 3 m chaetae; Th III with 1 a and 3 m chaetae; Abd I with 5 a, 4 m and 1 p chaetae, respectively. Three chaetae (1–3) on the upper side of bothriotrichum A, plus accessory a1 nearby its alveolus; b1 accessory chaeta between B and C bothriotricha, c2 just under C, c1 absent; bothriotricha A–C misaligned, with B bothriotrichum closer to C than A; dorso-posterior longitudinal series dI-1, dII-1, dIII-1 with 5–7, 9–10 and 6–8 chaetae, respectively; two rows with 3 chaetae each between C and D bothriotricha; D with 4 surrounding chaetae posteriorly; parafurcal area (furcula basis) with 8 regular chaetae; ventral complex with 1 chaeta (Fig. 3A). Small abdomen of the female: dorsal anal valve with as2–4, ms1–5?, mps1–3, and ps1–2 chaetae, ms1 and mps1–3 sword-shaped and smooth, 2 cuticular spines surrounding mps2; ventral anal valves each with ai1–6, ami1–2, mi1–5, mpi1–2, and pi1–3 chaetae, mi3 and mpi1–2 sword-shaped and smooth, mi5 as the subanal appendage long (similar in length to mi3, mpi1–2) with a spatulated and apically serrated apex (sometimes one of the lateral edges is also distally serrated), curved toward the genital opening, 2 cuticular spines surrounding mpi2 (Fig. 3B). Genital plate of the female unclear.

Abdominal appendages (Fig. 3C–F) Collophore with 1 distal chaeta on each side, with a pair of a little wrinkled, almost smooth, sacs. Tenaculum with 3 teeth on each ramus plus the basal tubercle, with a single unpaired apical chaeta. Furcal size length in holotype: manubrium = 75 µm; dens = 113 µm; and mucro = 75 µm (ratio 1:1.5:1). Manubrium with 5 dorsal chaetae on each side, the most proximal thinner than the others (Fig. 3C); dens ventral (or anterior) formula from the apex to the basis as 3,2,1,1,1, all chaetae enlarged except for the most proximal one, (Fig. 3D); dens dorsally (or posteriorly) with 16 chaetae, 7 of them on the lateral edges of the more distal region more robust, almost spine-like (Fig. 3E). Mucro apically swollen with both edges serrated from the basis until almost the apex (Fig. 3F).

Legs. (Figs 3A, 4) Epicoxae, subcoxae and coxae I–III with 1,0,1/1,1,4/1,1,4 chaetae, respectively, coxae II–III with 1 long and 1 tiny chaeta each (Fig. 3A). Trochanters I–III with 4 chaetae each, II–III with 1 chaeta each modified as an anterior trochanteral organ (Fig. 4A–C). Femurs I–III with 13/13/14 chaetae respectively, of which 1/1/3 as reduced chaetae (Fig. 4A–C). Tibiotarsi without oval organs, tibiotarsus I region F with 3 chaetae (FPae, FPe, and FPpe), whorls I–V with 9,8,8,8,9 chaetae respectively, whorl I without clearly modified chaetae except for a larger dorsal one, whorl V with 2 ventro-distal chaetae (Fig. 4D). Tibiotarsus II region F with 3 chaetae (FPae, FPe, and FPpe), whorls I–V with 9,8,8,8,7 chaetae respectively, whorl I without clearly modified chaetae except for a slightly larger dorsal one, whorl V with 1 ventro-distal chaeta (Fig. 4E). Tibiotarsus III region F with 4 chaetae (FPae, FPe, FPpe, and FSa), whorls I–V with 9,8,8,8,7 chaetae respectively, whorl I without clearly modified chaetae except for a slightly larger dorsal one, whorl V with 1 ventro-distal chaeta (Fig. 4F). Foot complexes I–III with 2 pretarsal chaetae each, 1 anterior and 1 posterior; ungues (claws) without cavity or pseudonychia, but with an underdeveloped tunica covering about 2/3 up to 3/4 of the dorsal ungues, lateral lamellae apparently lacking teeth, each unguis with one internal tooth; unguis I slender, III broad (Fig. 4D–F). Unguiculi (empodia) never surpassing the ungues, unguiculus I almost reaching the apex of unguis I, unguiculi II–III clearly shorter; unguiculi I–II with one proximal internal tooth each, unguiculus III with 2 more distal teeth (Fig. 4D–F).

**Male.** Not found, species possibly parthenogenetic.

###### Etymology.

The new species was named after its short antenna (from Latin *brevi*- = short; *cornis* = “horns”, antennae).

###### Distribution and habitat.

The new species was collected and only recorded in Jilin Province, Changling County, at the Ecological Research Station for Grassland Farm (ERSGF). This region is characterized by a semi-arid continental monsoon climate, with cold, dry winters and warm, rainy summers. Annual mean temperature in the region ranges from 4.68 to 6.48 °C, and annual precipitation is 280 to 400 mm with about 70% falling in the June-August period (Changling County Climate Station, Jilin Province). Changling County is located at a transitional zone of cropping and grazing, with high economical potential. However, drastic environmental disturbances are happening in this region, like sand and dust storms, emergence of saline-alkali soils, and land over-utilization.

###### Remarks.

*Arrhopalitesbrevicornis* sp. nov. belongs to the *A.caecus* (Tullberg, 1871) group of species *sensu*Vargovitsh (2013), according to its ventral (or anterior) dental chaetotaxy (3, 2, 1, 1, 1). Currently, there are ten other Asian species of the genus which belong to this group: *A.antrobius* Yosii, 1954, *A.abchasicus* Vargovitsh, 2013, *A.caecus*, *A.coreanus* Park & Kang, 2007, *A.gul* Yosii, 1966, *A.macronyx* Vargovitsh, 2012, *A.minor* Park & Kang, 2007, *A.minutus* Yosii, 1970, *A.potapovi* Vargovitsh, 2015 and *A.pukouensis*, (Bellinger et al. 1996–2022; Vargovitsh 2012, 2013, 2015). *Arrhopalitesbrevicornis* sp. nov. can be distinguished from all of them especially by the unguiculus III with 2 internal teeth (0–1 in all other species). Also, the combination of antennae less than 2 times the size of the head, Ant IV without annulations, 1+1 eyes, dorsal head with 9 spines, 2+2 regular spines per side on the anal valves, circumanal chaetae without basal serrations, subanal appendage long and apically serrated, similar in length to mi3, mpi1–2, manubrium with 5+5 and dorsal dens with 16 chaetae is unique among the Asian species of the *caecus* group (see Table 1).

**Table 1.** Comparison between the Asian species of *Arrhopalites* from the *caecus* group.

Species	*A.antrobius*	*A.abchasicus*	*A.caecus*	*A.coreanus*	*A.gul*	*A.macronyx*	*A.minor* ﻿	*A.minutus* ﻿	*A.potapovi* ﻿	*A.pukouensis* ﻿	*A.brevicornis* sp. nov.
Distribution	Japan	Abkhazia	Cosmopolitan	S. Korea	S. Korea	Abkhazia	S. Korea	Japan	Russia	China	China
Cave species	Yes	Yes	No	No	Yes	Yes	No	No	No	No	No
Body size (mm)	1.3	0.9	up to 1.0	0.7	1.2	1.2	0.5	0.5	up to 0.88	up to 1.3	0.71–0.81
Color pattern	unpigmented	unpigmented or with dorsal spots	unpigmented or with dorsal spots	with dorsal spots	unpigmented or with dorsal spots	with dorsal spots	with lateral spots	unpigmented	unpigmented, only eyes pigmented or with dorsal spots	unpigmented	with dorso-lateral spots
Ant IV annulations	4	5–7	(-)	(-)	7	7–8	(-)	(-)	(-/+)5–6	(-)	(-)
Ant at least 2× longer than head	Yes	Yes	No	Yes	Yes	Yes	No	No	No	No	No
Head dorsal spines	?	9	6–13	10 (possibly more)	(+)	(-)	9	13	13	(-)	9
Eyes	0+0	1+1	1+1	1+1	1+1	0+0?	1+1	1+1	1+1	0+0	1+1
Ungues I;II;III tunica	(+);(+);(+)	(–);(-);(-)	(-/+);(+);(+)	(+);(+);(+)	(-);(-);(-)	(-);(-);(-)	(+);(+);(+)	(-);(-);(+)	(-/+);(-/+);(-/+)	(-);(-);(-)	(+);(+);(+)
Ungues I–III inner tooth	(+)	(-/+)	(+)	(+)	(-/+)	(-)	(+)	(+)	(+)	(+)	(+)
Unguiculus III inner teeth	0	1	1	1	0–1	1	1	0	0–1	1	2
FAV cuticular spines (per side)	2+2	2+2 to 0+0	2+2	0+0	0+0	0+0	0+0	2+2	2+2(1+1 enlarged)	0+0	2+2
Subanal appendage shape	long, apically pointed	long, apically serrated	long, apically serrated	long, apically pointed	short, apically blunt	long, apically pointed	long, apically pointed	short, apically serrated	short, apically serrated	short, apically serrated	long, apically serrated
**ms1** chaeta shape	not forked	not forked	not forked	forked	not forked	not forked	not forked	not forked	not forked	not forked	not forked
Circumanal basally serrate chaetae	(-)	(+)	(+)	(-)	(+)	(+)	(-)	(-)	(+)	(-)	(-)
Tenaculum chaetae	1	1	1–2	1	1	1–2	1	?	1	1	1
Manubrium dorsal chaetae	?	5+5	?	5+5	4+4	5+5	9+9	4+4	5+5	5+5	5+5
Dorsal dens chaetae	more than 10	16	15?	16	16	16	14–16	14	15	15	16

Legends: Ant = antennal segment(s); S. = South; (-) = absent; (+) = present; (-/+) = absent or present;FAV = Female’s anal valves. Data based on the original descriptions, with the exception of *A.caecus* (based on Bretfeld 1999; Fjellberg 2007; and Vargovitsh 2013).

Concerning the species recorded from localities closer to Jilin Province, China, the South Korean *A.coreanus*, *A.gul* and *A.minor* share a similar color pattern, number of eyes, the presence of dorsal spines on head and number of dorsal dens chaetae with the new species. However the later differs from them by: the absence of Ant IV annulations (7 of *A.gul*); antennae less than 2 times the size of the head (at least two times in *A.coreanus* and *A.gul*); all ungues tunicate (without tunica on *A.gul*); female’s subanal appendage apically serrated (pointed in *A.coreanus* and *A.minor*, and blunt in *A.gul*); dorsal anal valve chaeta ms1 not forked (forked in *A.coreanus*); circumanal chaetae without basal serrations (with in *A.gul*) and manubrium with 5+5 chaetae (4+4 in *A.gul*, 9+9 in *A.minor*).

The only other species of the *caecus* group registered from China is *A.pukouensis*, from Nanjing, Jiangsu District, approximately 1800 km distant from the type location of the new species. Both species are vastly different as *A.pukouensis* is unpigmented (vs. pigmented), has no eyes and dorsal head spines (vs. 1+1 eyes and 9 spines, respectively), its ungues are devoid of tunica (*vs.* present); its female’s anal valves have no cuticular spines and their subanal appendage is short (vs. 2+2 spines per side and the subanal appendage is long, respectively) and its dorsal dens shows 15 chaetae (vs. 16 in the new species). A detailed comparison of the morphology and the known distribution of all the cited species is presented in Table 1. We also provide a key of all Asian species of *caecus* group below.

### ﻿Identification key to the Asian species of *caecus* group

**Table:** 

1	Females’ subanal appendage pointed	**2**
–	Females’ subanal appendage blunt or apically serrated	**5**
2	Ungues without tunica; at least part of the circumanal chaetae of females basally serrate	***A.macronyx* Vargovitsh, 2012**
–	Ungues with tunica; all circumanal chaetae of females basally smooth	**3**
3	Eyes absent; unguiculus III without inner teeth; female’s anal valves with 2+2 cuticular spines per side (Fig. 3B)	***A.antrobius* Yosii, 1954**
–	Eyes 1+1; unguiculus III with one inner tooth; female’s anal valves lacking cuticular spines	**4**
4	Dorsal head with 10 or more spines; dorsal anal valve ms1 chaeta forked; manubrium with 5+5 dorsal chaetae	***A.coreanus* Park & Kang, 2007**
–	Dorsal head with 9 spines; dorsal anal valve ms1 chaeta not forked; manubrium with 9+9 dorsal chaetae	***A.minor* Park & Kang, 2007**
5	Antennae at least two times longer than head length	**6**
–	Antennae shorter, less than two times the head length	**7**
6	Body size about about 0.9 mm; manubrium with 5+5 dorsal chaetae	***A.abchasicus* Vargovitsh, 2013**
–	Body size about about 1.2 mm; manubrium with 4+4 dorsal chaetae	***A.gul* Yosii, 1966**
7	Eyes absent; ungues III without tunica; female’s anal valves without cuticular spines	***A.pukouensis* Wu & Christiansen, 1997**
–	Eyes 1+1; ungues III with tunica; female’s anal valves with 2+2 cuticular spines per side	**8**
8	Female’s anal valves with 1+1 large modified cuticular spines per side	***A.potapovi* Vargovitsh, 2015**
–	Female’s anal valves with only small cuticular spines (Fig. 3B)	**9**
9	Unguiculus III with one inner tooth; female’s anal valves circumanal chaetae basally serrate	***A.caecus* (Tullberg, 1871)**
–	Unguiculus III toothless or with two inner teeth; female’s anal valves circumanal chaetae basally smooth	**10**
10	Dorsal head with 13 spines; ungues I–II without tunica; unguiculus III toothless; manubrium with 4+4 dorsal chaetae; dorsal dens with 14 chaetae	***A.minutus* Yosii, 1970**
–	Dorsal head with 9 spines; ungues I–II with tunica; unguiculus III with 2 inner teeth; manubrium with 5+5 dorsal chaetae; dorsal dens with 16 chaetae	***A.brevicornis* sp. nov.**

## ﻿Discussion

The current knowledge on the Chinese Symphypleona is still incipient, despite the recent efforts from different research groups in describing the local springtail fauna and studying its systematics. So far only 17 species of the order were recorded from China, mostly from dicyrtomids of the genera *Papirioides* Folsom, 1924 (6 spp.) and *Ptenothrix* Börner, 1906 (5 spp.) (Folsom, 1924; Denis, 1929; Lin and Xia, 1985; Itoh and Zhao, 1993; Chen and Christiansen, 1996; Guo and Chen, 1996; Wu and Chen, 1996; Li et al. 2007). The other records are from Arrhopalitidae (3 spp., including *A.brevicornis* sp. nov.) and Sminthuridae (2 spp.) and there is a single species of Bourletiellidae (Lin and Chen 1997; Wu and Christiansen 1997; Li et al. 2008; Chen et al. 2019). Due to the vast area of the country and its many different terrestrial habitats, it is likely these numbers are very far from representing the real richness of the Symphypleona from China, and further efforts should be done to better comprehend this particular fauna. Also, adequate strategies to manage the grazing intensity in Chinese grasslands are crucial to preserve endemic species from these regions.

## Supplementary Material

XML Treatment for Arrhopalites brevicornis
